# Transcriptional regulation of Annexin A2 promotes starvation-induced autophagy

**DOI:** 10.1038/ncomms9045

**Published:** 2015-08-20

**Authors:** Kevin Moreau, Ghita Ghislat, Warren Hochfeld, Maurizio Renna, Eszter Zavodszky, Gautam Runwal, Claudia Puri, Shirley Lee, Farah Siddiqi, Fiona M. Menzies, Brinda Ravikumar, David C. Rubinsztein

**Affiliations:** 1 Department of Medical Genetics, Cambridge Institute for Medical Research, Wellcome/MRC Building, Addenbrooke's Hospital, Hills Road, Cambridge CB2 0XY, UK

## Abstract

Autophagy is an important degradation pathway, which is induced after starvation, where it buffers nutrient deprivation by recycling macromolecules in organisms from yeast to man. While the classical pathway mediating this response is via mTOR inhibition, there are likely to be additional pathways that support the process. Here, we identify Annexin A2 as an autophagy modulator that regulates autophagosome formation by enabling appropriate ATG9A trafficking from endosomes to autophagosomes via actin. This process is dependent on the Annexin A2 effectors ARP2 and Spire1. Annexin A2 expression increases after starvation in cells in an mTOR-independent fashion. This is mediated via Jun N-terminal kinase activation of c-Jun, which, in turn, enhances the trans-activation of the Annexin A2 promoter. Annexin A2 knockdown abrogates starvation-induced autophagy, while its overexpression induces autophagy. Hence, c-Jun-mediated transcriptional responses support starvation-induced autophagy by regulating Annexin A2 expression levels.

## Abstract (web summary)

Starvation triggers autophagy by inhibiting mTOR signalling. Here, Moreau *et al.* identify an alternative, transcriptional activation pathway: starvation-induced JNK signalling upregulates Annexin A2 transcription, which in turn enhances actin-dependent transport of Atg9 vesicles from endosomes to autophagosomes.

## Graphical abstract

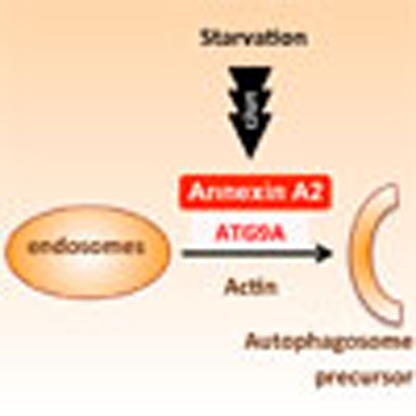

Macroautophagy is a conserved catabolic pathway in which cytosolic contents, such as damaged organelles, misfolded proteins and bacteria, are transported into lysosomes for degradation^1^,^2^,^3^. Autophagy plays an essential role in maintaining cellular homoeostasis, and deregulation of autophagy has been associated with a wide range of human conditions, including cancer, infection and neurodegeneration^1^,^2^,^3^. Autophagy is induced after starvation in organisms from yeast to man, where it buffers nutrient deprivation by degrading macromolecules. For example, this response is critical in the mammalian newborn period before the establishment of breastfeeding, where it protects against starvation^4^. The classical pathway regulating this process is mediated via inhibition of the (mammalian) target of rapamycin complex 1 (mTORC1), a kinase complex that acts on very early stages of autophagosome biogenesis^3^. However, as autophagosome formation likely requires membrane inputs from multiple routes^5^,^6^,^7^,^8^,^9^,^10^,^11^,^12^,^13^, it is possible that the starvation response needs to activate multiple nodes and may require the integration of additional signals for its maintenance. Indeed, autophagy is also regulated by transcription factors (for example, TFEB, FOXO3A and TFE3)^14^,^15^,^16^. However, these transcription factors appear to regulate a multitude of possible effector genes, and it is not clear which of the putative targets are necessary or sufficient to elicit the response. In some of these cases, there may well be multiple relevant target genes.

The membranes required for autophagosome formation seem to originate from multiple compartments, such as the endoplasmic reticulum, Golgi, mitochondria, plasma membrane, endoplasmic reticulum–Golgi intermediate compartment, and early and recycling endosomes^5^,^6^,^7^,^8^,^9^,^10^,^11^,^12^,^13^. The dynamics and the interactions that can occur between these compartments are still mysterious. Therefore, there is an urgent need to understand the trafficking of key autophagy proteins in greater detail. ATG9A is a transmembrane autophagy-related (ATG) protein that is thought to deliver membranes to the preautophagosome structures and autophagosomes^2^,^3^. Downregulation of ATG9A in yeast and mammalian cells inhibits autophagosome formation^17^,^18^,^19^,^20^,^21^,^22^,^23^. In mammalian cells, ATG9A traffics between Golgi, diverse endocytic vesicles and autophagosomes^13^,^17^,^18^,^24^. Recently, we found that ATG9A was routed from the plasma membrane to recycling endosomes via early endosomal compartments and this trafficking is important for autophagosome biogenesis^11^. While ATG9A traffics through the secretory pathway, most of the localization previously reported to be in the Golgi is probably due to its predominant localization in recycling endosomes^11^, which cannot easily be distinguished from the Golgi using static experiments^11^. Moreover, little is known about how ATG9A is sorted between these different compartments, which represents an important gap in the understanding of autophagosome biogenesis and its regulation. Here we show that actin is localized around ATG9A vesicles in mammalian cells and this is important for ATG9A sorting from endosomes. We identified three actin-nucleating factors, Annexin A2, ARP2 and Spire1 as important players in ATG9A sorting. Annexin A2 levels appear to be upregulated upon starvation in a Jun N-terminal kinase(JNK)-c-Jun-dependent manner, and this correlates with an increase in ATG9A vesicle movement and autophagosome formation.

## Results

### Annexin A2 regulates autophagy

**Figure 1:**
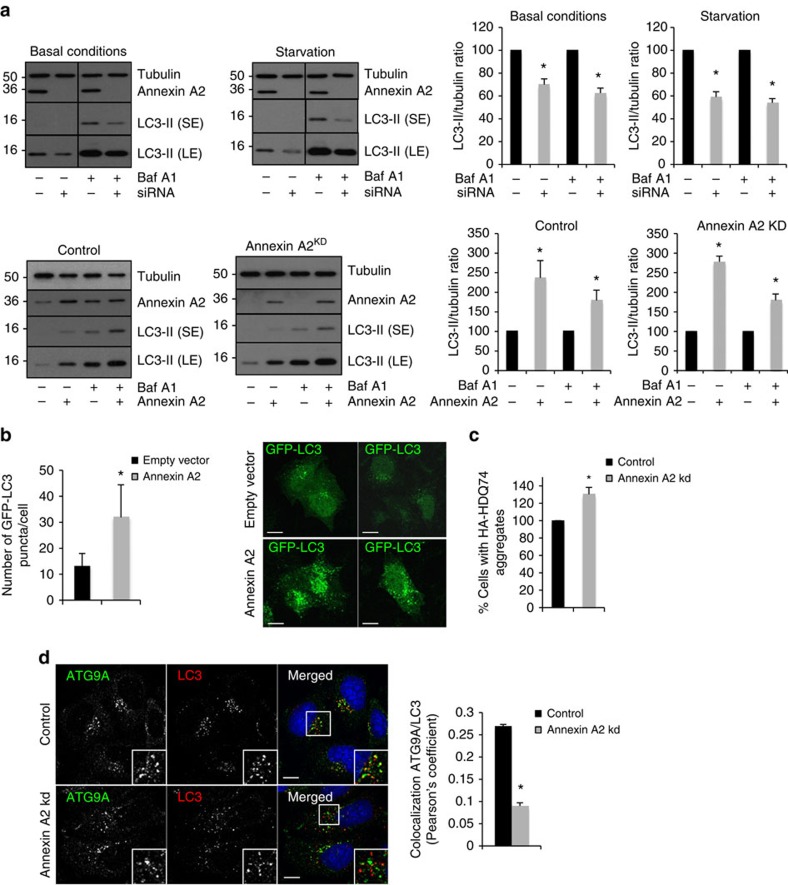
Annexin A2 regulates autophagy. (**a**) Western blot analysis of tubulin, Annexin A2 and LC3-II in HeLa where Annexin A2 was knocked down using siRNA and/or transiently expressed, as indicated. The cells were starved in HBSS and treated with bafilomycin A1 (BafA1) as indicated. (SE, short exposure; LE, longer exposure). Quantification of LC3-II/tubulin ratio is shown as mean ± s.e.m. (*P<0.05; two tail one-sample *t*-test). (**b**) Number of GFP-LC3 dots per cell in Annexin A2 transiently expressing cells. HeLa cells where Annexin A2 was transiently expressed for 24 h were fixed and subjected to microscopy. The data represent the number of GFP-LC3 dots per cell shown as mean ± s.d. (*P<0.05; two-tailed *t*-test; *n*≥50 cells per condition). Representative confocal pictures are shown. Scale bars, 5 μm. (**c**) Quantification the proportion of mutant huntingtin (Q74)-expressing cells with aggregates (HA-HDQ74) in Annexin A2 knockdown cells. HeLa cells transiently expressing HA-HDQ74 were fixed and subjected to microscopy after HA-HDQ74 immunostaining using an anti-HA specific antibody. Representative pictures are shown. Data are mean ± s.d. of the percentage of cells with HA-HDQ74 aggregates (*n*=3 experiments; *P<0.05; two tail one-sample *t*-test). Typically, about 25% of the control cells have aggregates. We have normalised controls to 100% to enable statistics from multiple experiments, as the control numbers vary in independent experiments. (**d**) Colocalization between ATG9A and LC3 in Annexin A2 knockdown HeLa cells upon starvation. Confocal pictures are presented with magnified areas showing the colocalization between ATG9A and LC3. Quantification of ATG9A and LC3 colocalization is shown on the right as Pearson's coefficient. Data are mean±s.e.m. (*n*≥20 cells; *P<0.05; two tail one-sample *t*-test). Scale bars, 5 μm.

As part of ongoing efforts to identify regulators of autophagy, we focussed on Annexin A2. Annexin A2 is an actin-binding protein that modulates many intracellular trafficking events via the regulation of actin polymerization^25^. Annexin A2 knockdown prevents endocytic transport beyond early endosomes^26^, effects that are mimicked by actin depolymerisation^27^. We tested if Annexin A2 was involved in autophagy using western blots to measure LC3-II levels. During autophagy, cytosolic LC3 is cleaved by ATG4 to form LC3-I, which can be conjugated to phosphatidylethanolamine to form LC3-II specifically on autophagosomal membranes^28^. Therefore, the level of LC3-II positively correlates with the number/volume of autophagosomes present inside the cell. However, an accumulation of LC3-II levels by western blot can either be the consequence of increased autophagosome formation, or impaired autophagosome degradation^29^. To discriminate between these two possibilities, the assay can be performed in the presence of saturating concentrations of Bafilomycin A1 (BafA1), a potent inhibitor of the vacuolar H^+^ ATPase that inhibits the degradation of autophagosomes^29^,^30^. Annexin A2 knockdown, using different short interfering RNAs (siRNAs), decreased LC3-II levels in the absence and in the presence of BafA1 in basal or starvation conditions (Fig. 1a and Supplementary Fig. 1a). Annexin A2 knockdown also decreased the number of GFP-LC3 dots (autophagosomes) per cell (Supplementary Fig. 1b), suggesting that Annexin A2 regulated autophagosome formation. Annexin A2 overexpression stimulated autophagy, as seen by an increase of LC3-II levels in the absence and in the presence of BafA1 by western blot (Fig. 1a) and by an increase of LC3 vesicles by immunofluorescence (Fig. 1b). Moreover, the autophagosome formation defect caused by Annexin A2 knockdown could be rescued by overexpressing Annexin A2 (Fig. 1a), confirming that the effects of Annexin A2 knockdown seen were not the consequence of off-target effects of the siRNAs. The inhibition of autophagosome formation when Annexin A2 was downregulated was associated with the accumulation of autophagic substrates like p62, and the proportion of mutant huntingtin (Q74)-expressing cells with aggregates (mutant huntingtin with 74 glutamine repeats is an autophagic substrate and the proportion of cells with aggregates is a function of its expression levels and correlates inversely with autophagic activity) (Fig. 1c and Supplementary Fig. 1c)^30^,^31^. On the other hand, Annexin A2 overexpression decreased p62 levels (Supplementary Fig. 1d).

Annexin A2 can form a heterotetrameric complex, consisting of two Annexin A2 molecules bound via their N termini to a dimer of p11/S100A10 light chains^25^. The light chain, and thus presumably formation of the heterotetramer, was reported to control Annexin A2 association with the plasma membrane and to cortical actin^25^,^32^. To test if the effect of Annexin A2 knockdown was a consequence of its actions at the plasma membrane, we knocked down the S100A10 light chain and found that this had no effect on either LC3-II levels or on the numbers of cells with Q74 aggregates (Supplementary Fig. 1e,f). These data suggest that Annexin A2-regulated autophagy via its location to another organelle, rather than the plasma membrane. For example, p11/S100A10 is not required for the association of Annexin A2 to endosomes or for its roles in early-to-late endosome trafficking^32^. Recent studies place Annexin A2 upstream of the actin-nucleating proteins ARP2 and Spire1 in the actin-dependent regulation of endosome maturation^27^. ARP2 and Spire1 knockdown decreased LC3-II levels in the presence of BafA1 (Supplementary Fig. 1g,h). Spire1 knockdown also reduced the number of GFP-LC3 dots per cell (Supplementary Fig. 1i). These data suggested that Annexin A2 may regulate autophagosome formation at the endosomal level by actin, potentially via ATG9A sorting. This hypothesis is consistent with the roles of Annexin A2 in actin-dependent endosomal trafficking^27^ and the need for ATG9A to traffic from early to recycling endosomes to the site of autophagosome biogenesis^11^. The actin cytoskeleton has been shown to play a role in ATG9A vesicle motion in yeast and *Drosophila*^33^,^34^,^35^, and a role for actin in autophagosome formation is supported by our recent data showing a role for the WASH complex in mammalian autophagy^36^. Our enthusiasm for a role of Annexin A2 in the endosomal trafficking of ATG9A was increased by the observation that Annexin A2 knockdown decreased the colocalization of ATG9A and LC3, suggesting that ATG9A was not able to reach autophagosomal membranes (Fig. 1c). Consistent with a defect in ATG9A trafficking, Annexin A2 knockdown decreased the level of the ATG5-12 and ATG16L1, which were previously shown to be downregulated as a consequence of ATG9A knockdown^18^,^37^, but did not decrease Beclin-1 or VPS34 levels (two autophagy proteins acting in the early steps of autophagosome biogenesis; Supplementary Fig. 1c).

### Annexin A2 regulates ATG9A sorting from endosomes

**Figure 2:**
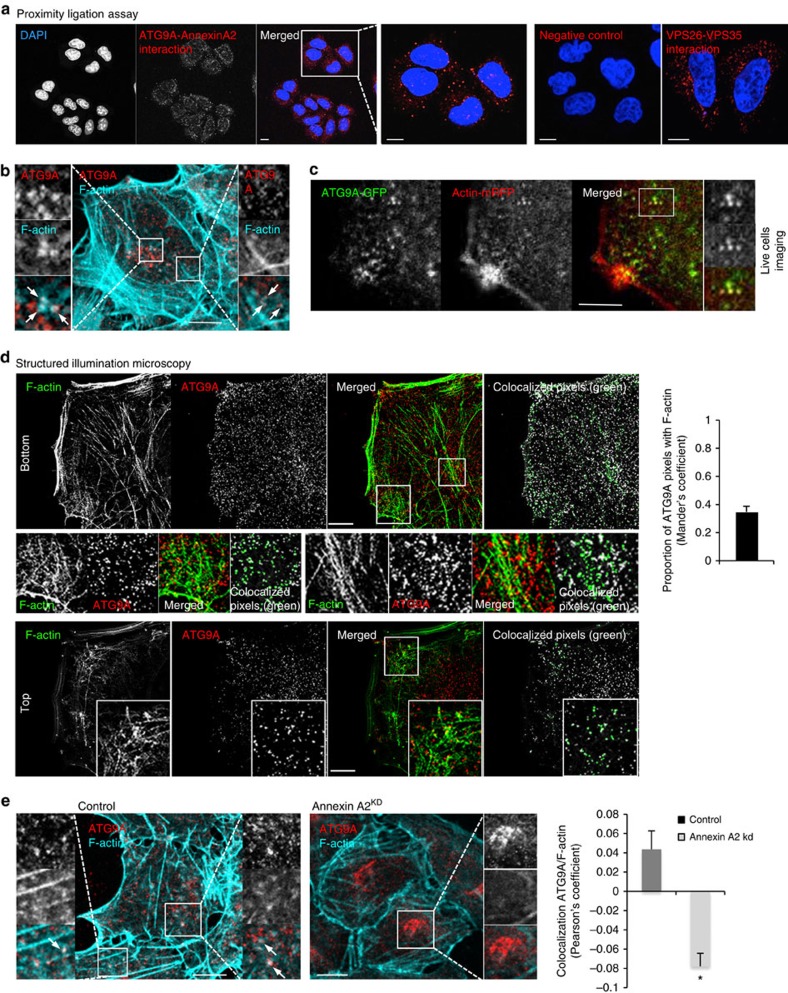
Annexin A2 regulates ATG9A and actin colocalization. (**a**) HeLa cells were fixed and analysed using the Proximity Ligation Assay, with primary antibodies as indicated. The cells were imaged by confocal microscopy. Scale bars, 5 μm. (**b**) Colocalization between ATG9A and F-actin in HeLa cells. Confocal pictures showing colocalization between endogenous ATG9A and F-actin (using Phalloidin staining) are presented. Magnified areas are shown with arrows indicating actin patches around ATG9A vesicles. Scale bars, 5 μm. (**c**) Colocalization between ATG9A and actin in live cells. Confocal pictures showing colocalization between endogenous ATG9A and actin (using actin-mRFP) are presented. Magnified areas are presented showing ATG9A and actin colocalization. Scale bars, 5 μm. See Supplementary Movie 1. (**d**) Colocalization between ATG9A and F-actin by structured illumination super-resolution microscopy. Cells were fixed, immunostained for endogenous ATG9A and F-actin using Phalloidin conjugated to Alexa555 and subjected to structured illumination microscopy. Top and bottom show slices through the top and bottom of the cells, respectively. Colocalized pixels are shown in green on the right of each panel using ImageJ. Colocalization between ATG9A and F-actin is shown as Manders' coefficient representing the number of ATG9A pixel colocalizing with F-actin pixels. Data are mean ± s.e.m. Scale bars, 5 μm. (**e**) Colocalization between ATG9A and F-actin (using Phalloidin staining) in Annexin A2 knockdown cells. Confocal pictures are presented with magnified areas showing the colocalization between ATG9A and F-actin in control cells and a decreased colocalization in Annexin A2 knockdown cells. Scale bars, 5 μm. Data are Pearson's coefficient as mean ± s.e.m. (*n*≥20 cells; *P<0.05; two tail *t*-test).

The likelihood that Annexin A2 directly regulates the trafficking of ATG9A-containing endosomes was supported by the observation of a pool of ATG9A in close proximity to Annexin A2 as shown by proximity ligation assay (Fig. 2a; the positive control used VPS26–VPS35 proteins, part of retromer complex). We also observed colocalization between endogenous ATG9A and endogenous Annexin A2 by confocal microscopy and electron microscopy (Supplementary Fig. 2a,b). As Annexin A2 serves as an anchor for nucleating actin polymerisation on endosomes^27^, we analysed the colocalization between endogenous ATG9A and filamentous F-actin using phalloidin toxin (an F-actin-binding toxin) conjugated to Alexa488 or by expressing mCherry-Lifeact-7 protein^38^. As seen in Fig. 2b and Supplementary Fig. 2c,d, F-actin formed patches around ATG9A vesicles and some ATG9A vesicles with actin patches were also positive for LC3. We observed the localization of actin on ATG9A vesicles by live cell imaging using transient expression of ATG9A-GFP and actin-mRFP (Fig. 2c and Supplementary Movie 1). We confirmed the localization of actin on ATG9A vesicles by super-resolution-structured illumination microscopy and found that >30% of ATG9A vesicles colocalized with actin (Fig. 2d). Since not all ATG9A vesicles colocalized with actin, we next analysed the localization of actin-positive ATG9A vesicles with an endosomal marker, given the previous literature on the role of actin in endosomal maturation^27^,^39^, and our previous demonstration of trafficking of ATG9A through early endosomes^11^. We observed the presence of Annexin A2 on ATG9A vesicles positive for EEA1 (an early endosomal maker) or actin (Supplementary Fig. 2e). The localization of actin around ATG9A vesicles was decreased by Annexin A2 knockdown, suggesting that Annexin A2 serves as an anchor for actin on ATG9A vesicles (Fig. 2e). These effects parallel previous observations that Annexin A2 impairs actin patch nucleation on endosomes^27^. (Note that the Pearson's coefficient is derived from a correlation analysis, and is expected to be low for F-actin and ATG9A, since while a fair proportion of the ATG9A is associated with F-actin, only a small percentage of the F-actin pixels colocalizes with ATG9A, as F-actin is widely distributed with a major component being at the cell cortex.)

**Figure 3:**
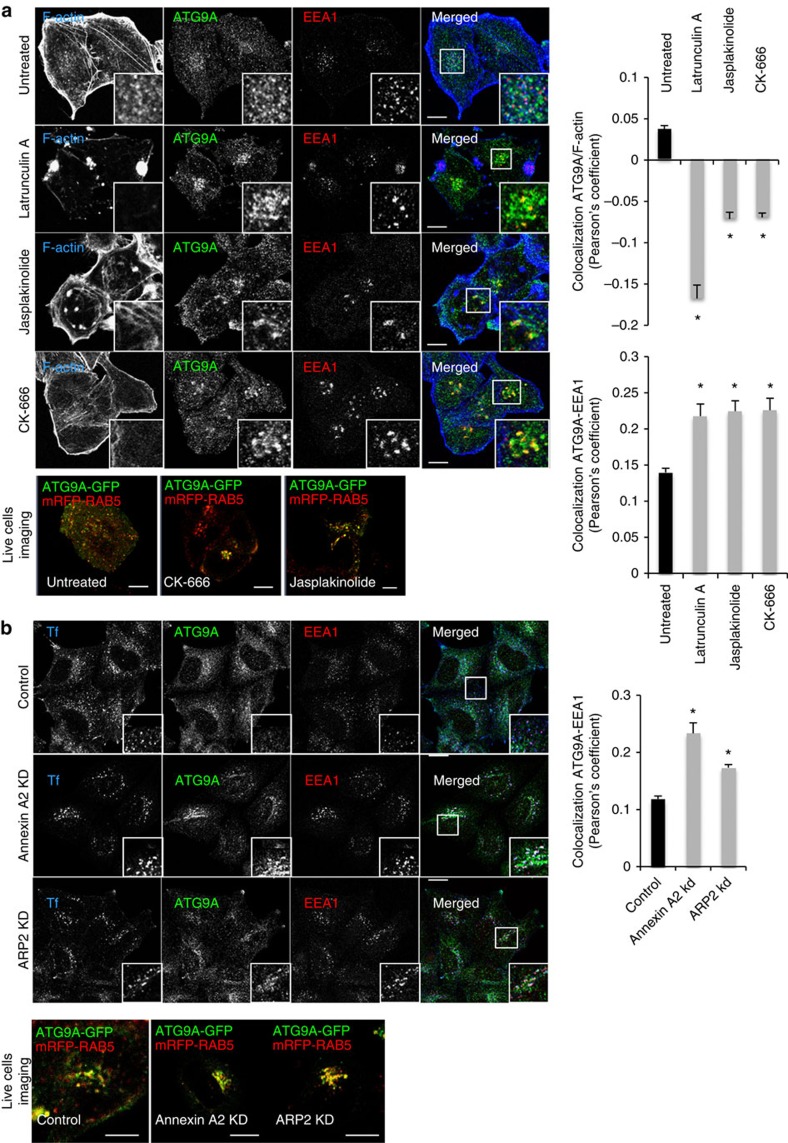
Actin and Annexin A2 regulate ATG9A sorting from endosomes. (**a**) Colocalization between ATG9A, EEA1 and F-actin in HeLa cells treated with drugs affecting actin polymerization for 2 h; latrunculin A (0.5 μM), jasplakinolide (200 nM) and CK-666 (50 μM). Confocal pictures showing colocalization between ATG9A, EEA1 and F-actin (using Phalloidin staining) are presented with magnified areas. Colocalization between ATG9A and RAB5 (using mRFP-RAB5) in live cells is also shown (Supplementary Movies 2–4). Quantification of ATG9A with F-actin and ATG9A with EEA1 colocalization is shown on the right as Pearson's coefficient. Data shown as mean ± s.e.m. (*n*≥20 cells; *P<0.05; two tail *t*-test). Scale bars, 5 μm. (**b**) Colocalization between ATG9A, EEA1 and internalized transferrin in Annexin A2 and ARP2 knockdown cells. Confocal pictures showing colocalization between ATG9A, EEA1 and internalized transferrin (30 min) are presented with magnified areas. Colocalization between ATG9A and RAB5 (using mRFP-RAB5) in live cells is also shown (Supplementary Movies 5–7). Quantification of ATG9A and EEA1 colocalisation is shown on the right as Pearson's coefficient. Data are mean ± s.e.m. (*n*≥20 cells; *P<0.05; two tail *t*-test). Scale bars, 5 μm.

To test the role of actin in ATG9A trafficking, we used drugs that depolymerize (latrunculin A: LatA and CK-666 (an ARP2/3 inhibitor)) or immobilize (Jasplakinolide: Jak) the actin cystokeleton^40^,^41^,^42^. In cells treated with Latrunculin A, CK-666 and jasplakinolide, we observed a decrease in ATG9A-actin colocalization, an increase in ATG9A-early endosome (EEA1) colocalization (Fig. 3a) and an increase in ATG9A-RAB5 (a marker of early endosomes) colocalization as seen by live cell imaging (Fig. 3a and Supplementary Movies 2–4). Moreover, inhibition of ARP2 via CK-666 treatment decreased the localization of actin to ATG9A vesicles positive for EEA1, but did not affect the recruitment of Annexin A2 to ATG9A vesicles (Supplementary Fig. 2e). Annexin A2 and ARP2 knockdown recapitulated the phenotype observed with actin drugs—an increased colocalization between ATG9A and EEA1 or RAB5 (Fig. 3b and Supplementary Movies 5–7). Moreover, the size of endosomes (EEA1-positive) increased like in Annexin A2 and ARP2 knockdown cells (Fig. 3b), consistent with impaired trafficking out of the early endosomes. Consistent with this model, transferrin, which normally traffics from early endosomes to recycling endosomes, accumulated in the ATG9A-EEA1 positives vesicles in Annexin A2 and ARP2 knockdown cells (Fig. 3b). These data suggest that ATG9A sorting from early endosomes requires an Annexin A2-dependent actin mechanism. To assess if this disturbed ATG9A trafficking was functionally relevant to autophagy, we tested if these actin-perturbing drugs affected autophagosome formation. As seen in Supplementary Fig. 3a–d, Latrunculin A, CK-666 and Jasplakinolide increased LC3-II in the absence of BafA1, but decreased LC3-II in the presence of BafA1 and decreased ATG9A and LC3 colocalization. These data suggest that these drugs play a role in both autophagosome formation (LC3-II levels with BafA1) and autophagosome degradation (LC3-II levels without BafA1). The latter could be the consequence of the role of actin in the recycling of the vATPase pump, as shown recently^43^. The effect of these drugs on autophagosome formation may be due to defective ATG9A sorting from endosomes. Given that the levels of LC3-II in Annexin A2 knockdown cells decreased with or without BafA1 (Fig. 1a), this suggests that Annexin A2 does not play a role in autophagosome clearance. The effect of Annexin A2 downregulation on ATG9A sorting could be rescued by transiently expressing Annexin A2, as seen by the reduction of the early endosomes size (EEA1 vesicles), ATG9A clustering and ATG9A-EEA1 colocalization (Supplementary Fig. 3e).

### Annexin A2 regulates ATG9A sorting to recycling endosomes

**Figure 4:**
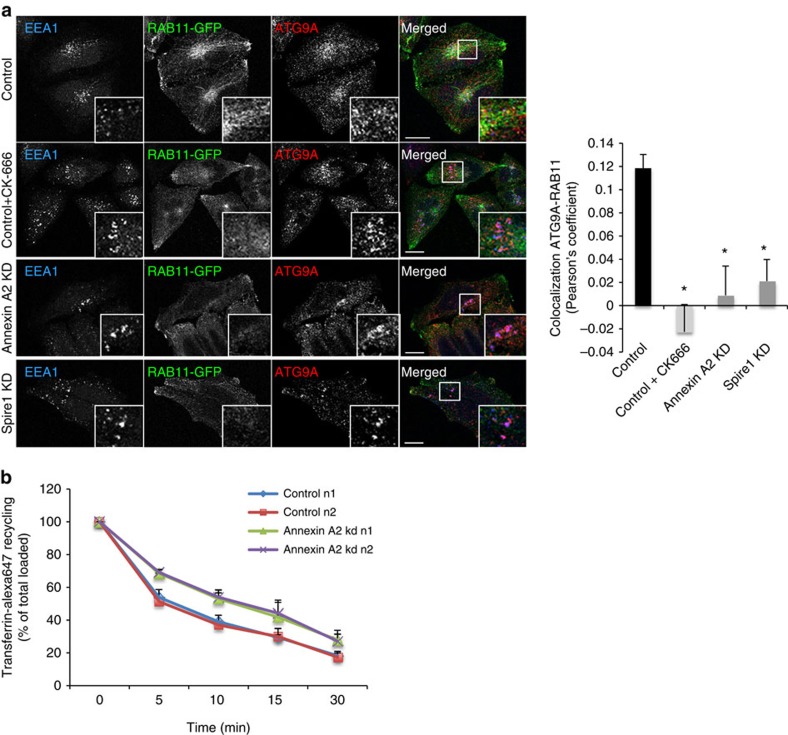
Actin and Annexin A2 regulate ATG9A localization to recycling endosomes. (**a**) Colocalization between ATG9A, EEA1 and RAB11-GFP in HeLa treated with CK-666 (50 μM) drug affecting actin polymerization for 2 h or knockdown for Annexin A2 and Spire1 for 72 h. Confocal pictures showing colocalization between ATG9A, EEA1 and RAB11-GFP are presented with magnified areas. Quantification of ATG9A and RAB11 colocalization is shown on the right as Pearson's coefficient. Data shown as mean ± s.e.m. (*n*≥20 cells; *P<0.05; two tail *t*-test). Scale bars, 5 μm. (**b**) Cells knocked down for Annexin A2 were subjected to transferrin recycling assay using flow cytometry. Data show transferrin-Alexa647 recycling represented as the percentage of previously endocytosed transferrin remaining in the cells over time. This is higher in the Annexin A2 knockdown cells, thus, Annexin A2 knockdown retards recycling. Data shown as mean ± s.e.m. (*n*=2 experiments performed in duplicate).

We and others recently showed that ATG9A localizes transiently to recycling endosomes, which play an important role in autophagosome biogenesis. In order to understand if Annexin A2 regulates ATG9A sorting from early endosomes to recycling endosomes, we knocked down Annexin A2 and Spire1 or inhibited actin polymerization with CK-666 treatment. We first confirmed that a pool of ATG9A vesicles is associated with recycling endosomes using RAB11 as a marker (Fig. 4a). Annexin A2 and Spire1 knockdown or CK-666 treatment decreased the localization of ATG9A to RAB11-positive vesicles but increased its localization to EEA1-positive structures, as seen previously (Fig. 4a). Consistent with this colocalization analysis, the recycling of transferrin is impaired in Annexin A2 knockdown cells (Fig. 4b). These data suggest that Annexin A2 regulates ATG9A trafficking from early to recycling endosomes.

### Starvation upregulates Annexin A2 expression via JNK-c-Jun

**Figure 5:**
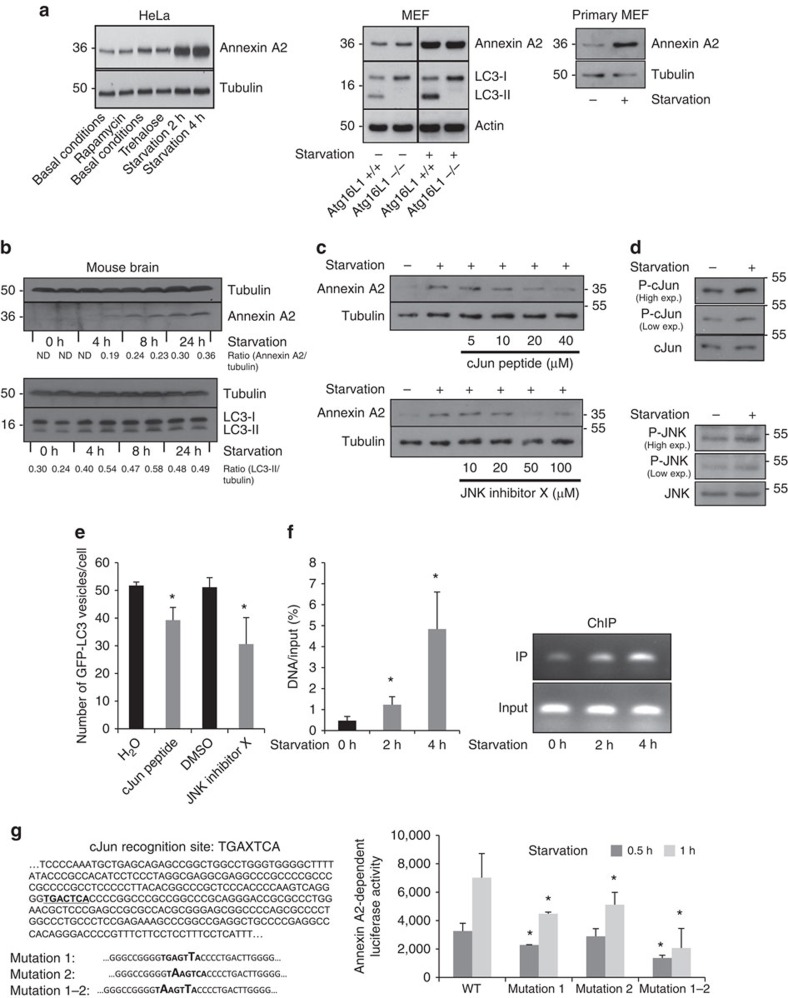
Starvation upregulates Annexin A2 expression via JNK. (**a**) Western blot analysis of tubulin or GAPDH, Annexin A2, LC3-II and actin in HeLa cells, immortalized MEF cells (ATG16L1 +/+ and ATG16L1 −/−) and primary MEF cells. The cells were starved in HBSS as indicated. (**b**) Western blot analysis of tubulin, Annexin A2 and LC3-II in mouse brain. Mice were starved for different time points as indicated. Quantifications of Annexin A2/tubulin and LC3-II/tubulin ratios are shown under the blots. (**c**) Western blot analysis of Annexin A2 and tubulin. HeLa cells were starved in HBSS for two hours and treated with c-Jun peptide or JNK inhibitor X at the concentrations indicated. (**d**) Western blot analysis of total and phospho-c-Jun at Ser 63 and 73 (upper panel) and total and phospho-JNK at Thr183 and Tyr185 (lower panel). HeLa cells were starved in HBSS for 2 h. (**e**) HeLa cells stably expressing GFP-LC3 were starved in HBSS and treated with c-Jun peptide (40 μM) or JNK inhibitor X (100 μM) for two hours. Then cells were fixed and subjected to automatic counting of LC3 vesicles. Quantification of autophagosomes (GFP positive vesicles)/cell is shown. At least 2,000 cells were counted per experiment; Data are mean ± s.d. (*n*=3 experiments; *P<0.05; two tail one-sample *t*-test). (**f**) HeLa cells starved in HBSS were subjected to a ChIP assay. The amount of *in vivo* binding of endogenous c-Jun to the promoter of ANNEXIN A2 was quantified by real-time PCR. Data are mean ± s.d. (*n*=3 experiments; *P<0.05; two tail *t*-test). (**g**) A part of the sequence of the Annexin A2 promoter is shown on the left. It contains the specific binding site of c-Jun that is highlighted in bold. The DNA binding consensus sequence is TGAXTCA, X can be G or C. Point mutations and a double mutation were introduced within the c-Jun recognition site. HeLa were transfected for 48 h with a luciferase reporter containing the Annexin A2 promoter without (WT) or with the indicated mutations. The cells were starved in HBSS. Data are mean ± s.d. of Annexin A2-dependent luciferase activity (*n*=3 experiments; *P<0.05 relative to the corresponding WT; two-tail *t*-test).

Amino acid and serum starvation is a classical autophagy-inducing stimulus^3^,^44^, which increased ATG9A movement in an actin-dependent manner, as seen by the automatic tracking of ATG9A vesicles using live cell imaging software, since the movement was abrogated by jasplakinolide (Supplementary Fig. 4a,b). The increase of ATG9A vesicle movement upon starvation could be mimicked by overexpressing Annexin A2 in cells grown in fed conditions and was reversed in Annexin A2 or ARP2 knockdown cells (Supplementary Fig. 4a,b). Starvation also upregulated Annexin A2 expression at the protein level in HeLa cells, immortalized mouse embryonic fibroblasts (MEFs) and primary mouse fibroblasts (primary MEF; Fig. 5a and Supplementary Fig. 4c). Upregulation of Annexin A2 protein levels was autophagy independent, as this occurred in ATG16L1 knockout cells (that do not have any LC3-II; Fig. 5a). Starvation increased Annexin A2 mRNA levels (Supplementary Fig. 4d). The effect of starvation on Annexin A2 expression seemed to be independent of TFEB, described as a positive transcription factor regulating autophagy upon starvation^14^, as we did not observe an increase in Annexin A2 level when TFEB was overexpressed, even in its constitutively active form (Supplementary Fig. 4e). TFEB knockdown also did not affect starvation-induced Annexin A2 levels (Supplementary Fig. 4e). Interestingly, starvation increased Annexin A2 and LC3-II levels in mouse brains (Fig. 5b). Before starvation, Annexin A2 levels were very low in the brain. This is consistent with the literature showing that Annexin A2 is expressed at low levels in brain and is upregulated in response to stresses such as tumours, inflammation and neurodegeneration^45^. Starvation represents a stress for the brain that needs a constant supply of energy, suggesting that starvation-dependent autophagy in the brain may represent an important pathway to maintain brain homoeostasis, and Annexin A2 could be an important regulator of this pathway.

Using the Text Mining Application from SABiosciences and the UCSC Genome Browser, a binding site for c-Jun and AP-1 transcription factors in the promoter of Annexin A2 was predicted. Therefore, we examined whether c-Jun was involved in the upregulation of Annexin A2 under starvation. The inhibition of c-Jun by pharmacological compounds (JNK inhibitor X) and a peptide that prevents c-Jun binding to its activator JNK (c-Jun peptide) abrogated the increase of Annexin A2 levels under starvation in a concentration-dependent manner (Fig. 5c and Supplementary Fig. 4f). During starvation, we observed phosphorylation of c-Jun at Ser 63 and 73, which is indicative of its activation by JNK^46^ (the transcriptional activity of c-Jun is strongly potentiated by phosphorylation at these sites) as well as the phosphorylation of JNK at Thr183 and Tyr185 (Fig. 5d). The JNK inhibitor X c-Jun peptide decreased starvation-induced autophagy, as measured by the decrease of the number of LC3 vesicles per cell LC3-II (Fig. 5e). Also, starvation increased the amount of c-Jun binding to Annexin A2 promoter, as assessed by chromatin immunoprecipitation (ChIP; Fig. 5f). Moreover, starvation enhanced the transcriptional activation of Annexin A2 promoter, whereas point mutations in c-Jun binding site within Annexin A2 promoter significantly reduced this effect and a double mutation decreased it even more dramatically (Fig. 5g). These results support a role of c-Jun in the upregulation of Annexin A2 transcription under starvation.

## Discussion

Autophagy upregulation is essential for surviving periods of starvation. While the mTOR pathway is a key conserved mechanism enabling this response, it regulates the ULK1/2–ATG13 complex, which acts far upstream in the process of autophagosome biogenesis^47^. Another pathway that is regulated after starvation in cells is an increase in Beclin-1 activity mediated by JNK phosphorylation of Bcl-2, which in its unphosphorylated state binds and inhibits Beclin-1 (ref. 48). Here we describe a transcriptional response pathway regulating autophagy, which is signalled by JNK. We find that autophagy upregulation in cells or mouse brain after starvation correlated with increased Annexin A2 levels. The starvation response was dependent on Annexin A2 levels as it was abrogated by Annexin A2 knockdown, while Annexin A2 overexpression in fed cells was sufficient to enhance autophagosome biogenesis. The induction of Annexin A2 was mTOR independent, and was mediated at the transcriptional level by JNK-activated c-Jun binding to the Annexin A2 promoter. The identification of multiple cooperating responses enabling maintenance of starvation-induced autophagy would be consistent with the notion that autophagosome formation requires multiple converging membrane trafficking pathways.

Annexin A2 regulates autophagy via its effects on actin and ATG9A trafficking. We have characterized the sorting of ATG9A from endosomes and identified actin and actin-nucleating factors such as Annexin A2, ARP2 and Spire1 as important players. Actin had previously been shown to be involved in autophagosome formation but the mechanism remained unclear, as it may affect multiple steps, including trafficking, recruitment of key autophagy proteins and autophagosome maturation^33^,^34^,^35^,^49^. We have observed that actin forms patches around ATG9A vesicles and the perturbation of actin polymerization (using drugs, or knockdown of actin-nucleating factors) trapped ATG9A in early endosomes and decreased its association with recycling endosomes and autophagosomes. Our study provides a mechanism of ATG9A sorting from endosomes via the control of actin dynamics by Annexin A2, ARP2 and Spire1. As this parallels the roles of these proteins in early-to-late endosomal trafficking^27^, and impaired exit of ATG9A from early endosomes will attenuate autophagosome biogenesis^11^, it is likely that the effects of Annexin A2 on ATG9A trafficking are sufficient to account for the impaired autophagosome formation. While Annexin A2 downregulation reduced the levels of ATG16 and ATG5/12, these effects were previously reported after ATG9A downregulation^18^,^37^ and while these would be consistent with the ATG9A-centric models we have proposed, we cannot exclude the possibility that these may be effects also mediated by ATG9A-independent consequences of Annexin A2 inhibition. For example, a recent student recently found that Annexin A2-impaired ATG16 vesicle biogenesis and homotypic fusion^50^.

In summary, we have identified Annexin A2 as a protein that is upregulated upon starvation via JNK and c-Jun. Annexin A2 upregulation is important for inducing autophagy, especially during starvation. This JNK-c-Jun-Annexin A2 pathway contrasts with previously described transcriptional pathways impacting on autophagy, like TFEB, TFE3 and FOXO3A, where single effector genes have not been identified^14^,^15^,^16^. The JNK-c-Jun-Annexin A2 axis may have relevance in disease contexts as well. Nutrient deprivation limits the growth of metastatic tumours and autophagy appears to be one way that can ameliorate this phenomenon, and may thus contribute to metastatic cancer growth. It is attractive to speculate about the potential anti-autophagic mechanisms for the beneficial effects of neutralizing Annexin A2 in treating cancer, since this appears to have efficacy in a human breast tumour xenograft model, where an Annexin A2-neutralizing antibody reduced tumour growth^51^,^52^.

## Methods

### Cell culture

HeLa cells (from ATCC) and MEF (immortalised, from Yoshimori Tamotsu, Osaka University, Japan or primary from Roger Davis, University of Mass. Medical School, USA) were cultured in DMEM D6546 (Molecular Probes) containing 10% fetal bovine serum, supplemented with 2 mM L-glutamine and 100 U ml^−1^ penicillin/streptomycin in 5% CO_2_ at 37 °C. HeLa cells stably expressing GFP-LC3 were cultured in DMEM D6546 containing 10% fetal bovine serum supplemented with 2 mM L-glutamine, 100 U ml^−1^ penicillin/streptomycin and 500 μg ml^−1^ G418 (Sigma) in 5% CO_2_ at 37 °C.

### Antibodies and reagents

Antibodies include: mouse monoclonal anti-tubulin (Sigma; T9026; 1/4,000), rabbit anti-LC3 for western blot (Novus Biologicals; NB100-2220; 1/4,000), mouse monoclonal anti-LC3 for immunofluorescence (MBL International; M152-3; 1/100), rabbit anti-GFP (Clontech; 632460; 1/1,000); rabbit anti-ATG9A (Abcam; EPR2450(2); 1/250); rabbit anti-actin (Sigma; A2066; 1/2,000); mouse monoclonal anti-Annexin A2 (BD Biosciences; 610071; used for wersternblot analysis; 1/1,000); mouse monoclonal anti-Annexin A2 (Santa Cruz Biotechnology; 3D5; used for proximity ligation assay; 1/100); mouse anti-EEA1 (Abcam; 1G11; 1/200), mouse anti-ARP2 (Abcam; 49674; 1/500); mouse anti-GAPDH (Abcam; ab8245; 1/4,000), rabbit anti-TFEB (Cell Signaling; 4240; 1/500), rabbit anti-ATG12 (Cell Signaling; 4180S; 1/1,000), rabbit anti-Beclin-1 (Cell Signaling; D40C5; 1/1,000), rabbit anti-Spire1 (Santa Cruz Biotechnology; sc-85162; 1/500), rabbit anti-VPS34 (Life Technologies; 38-2100; 1/1,000), rabbit anti-JNK (Cell signalling; 9258; 1/1,000), mouse anti-phospho JNK (Cell signalling; 9255; 1/1,000), rabbit anti-phospho-Jun (ser 63) (Cell Signaling; 9261S; 1/1,000), rabbit anti-Jun (Cell Signaling; 9162; 1/1,000) mouse anti-VPS35 (Santa Cruz Biotechnology, sc-374372; 1/500) and rabbit anti-VPS26 (Abcam; ab23892; 1/1,000), described previously^53^.

Reagents include: bafilomycin A1 (Sigma), Alexa Fluor 488-, 546-, 647-phalloidin (Invitrogen; A12379, A22283, A22287 respectively), latrunculin A (Sigma; L5163; used at 0.5 μM for 2 h), jasplakinolide (Sigma; J4580; used at 200 nM for 2 h), Alexa Fluor 488-transferrin (Invitrogen; T-11342), CK-666 (Sigma; SML-0006; used at 50 μM for 2 h), 420140 JNK Inhibitor X (Calbiochem; BI-78D3) and c-Jun peptide (Tocris; 1989).

### Plasmids

Annexin A2, ATG9A-GFP, TFEB-FLAG (wild type or S142A mutant) actin-mRFP, mRFP-RAB5, GFP-LC3 and mRFP-LC3 have been described previously^14^,^20^,^27^,^54^,^55^. mCherry-Lifeact-7 was a gift from Michael Davidson (Addgene plasmid # 54491). The luciferase reporter containing Annexin A2 promoter with c-Jun binding site was obtained from GeneCopoeia (Promoter reporter clone for Human NM_001002858, reference HPRM12525-PG02).

### Cell transfection

The cells were seeded at 1–2 × 10^5^ per well in 6-well plates and transfection was performed using LipofectAMINE or TransIT-2020 (for DNA) or LipofectAMINE 2000 (for siRNA and double transfections with DNA and siRNA; Invitrogen, Mirus), using the manufacturer's protocol. Pre-designed siRNA were ordered from Thermo Scientific (Dharmacon Technologies) (siRNA IDs: Annexin A2—ON-TARGETplus SMARTpool and Set of 4, L(U)-010741; ARP2—ON-TARGETplus SMARTpool, L-012076; Spire1—ON-TARGETplus SMARTpool, L-023397; TEFB—ON-TARGETplus SMARTpool and set of 4, LQ-009798) or Invitrogen (Annexin A2—s1385).

### Modulation of autophagy

To inhibit LC3-II degradation, cells were treated with Bafilomycin A1 diluted in cell culture media to a working concentration of 400 nM for 4 h, which is saturating for this effect^56^. To induce autophagy in an mTOR-dependent manner, cells were amino acid- and serum-starved in Hanks balanced salt solution (HBSS; Sigma) for 1–4 h.

### Food deprivation in mice

All animal studies were performed under the jurisdiction of appropriate Home Office Project and Personal animal licences and with local Ethics Committee approval from the University of Cambridge. All mice were weighed at the start of experiments. Twelve-week-old male C57BL/6 mice were deprived of food for 22.5 h, followed by free access to food for 1.5 h. All mice were weighed before (and also after) feeding to determine weight loss (15% or more would change the severity limit from mild to moderate, would result in schedule one killing of mice). Mice tissues were collected at different time point after feeding: immediately after giving food after 4 h, after 8 h and after 22.5 h. All mice had free access to water throughout the procedure.

### Western blotting

Cells were collected, rinsed with phosphate buffered saline (PBS), and lysed on ice for 30 min in PBS containing 1% Triton X100 and complete protease inhibitor cocktail (Roche). Lysates were centrifuged at 12,000*g* for 5 min at 4 °C, and supernatants were resolved by SDS–polyacrylamide gel electrophoresis and transferred to polyvinylidene difluoride membranes. The membranes were blocked with Tris-buffered saline 0.1% Tween-20 (TBST) containing 1% non-fat dry milk and were then incubated overnight at room temperature with primary antibodies diluted in TBST. Membranes were washed with TBST, incubated for 1 h at room temperature with 2,500 × dilutions of HRP-conjugated secondary antibodies (GE Healthcare Bioscience) in TBST containing 1% non-fat dry milk, and washed. Immunoreactive bands were then detected using ECL (GE Healthcare Bioscience). Uncropped scans for the most important experiments are shown in Supplementary Fig. 5.

### Fluorescence and immunofluorescence microscopy

For immunofluorescence microscopy, cells were cultured on coverslips, fixed with 4% paraformaldehyde in PBS for 5 min or with ice-cold methanol for 5 min, and permeabilized with 0.1% Triton X100 in PBS for 5 min. Coverslips were incubated with primary antibodies for 2 h, washed three times with PBS, and incubated with secondary antibodies for 30 min. Samples were mounted using ProLong Gold antifade reagent with or without DAPI (4,6-diamidino-2-phenylindole; Invitrogen) and observed using a Zeiss LSM710 laser confocal microscope. Samples were analysed by Structured Illumination Microscopy using an ELYRA Superresolution microscope from Zeiss. Automatic counting of LC3 vesicles from HeLa cells stably expressing GFP-LC3 was performed using the Cellomics ArrayScan VTI HCS Reader (20 × objective) and the Spot Detector V3 Cellomics BioApplication (Thermo Fisher Scientific). The numbers of vesicles per cell were counted for two thousand cells per coverslip and the mean number of vesicles per cell was calculated by the ArrayScan software.

### Immunogold electron microscopy

HeLa cells were fixed with a mixture of 2% paraformaldehyde and 0.2% glutaraldehyde in PBS for 2 h, at room temperature. Cells were then prepared for ultrathin cryosectioning and immunogold-labelled, as previously described^57^. Briefly, fixed cells were washed once in PBS/0.02 M glycine, after which cells were scraped in 12% gelatin in PBS and embedded in the same solution. The cell-gelatin was cut into 1 mm blocks, infiltrated with 2.3 M sucrose at 4 °C, mounted on aluminium pins and frozen in liquid nitrogen. Ultrathin cryosections were picked up in a mixture of 50% sucrose and 50% methyl cellulose and incubated with specific antibodies revealed with 10 or 15 nm protein A gold (Utrecht).

### Quantification of colocalization

Pearson's coefficient is a standard statistical analysis designed to measure the strength of a linear relationship between two variables. From a technical perspective the Pearson's coefficient is robust to issues like background and signal intensity. We used the Pearson's coefficient to analyze colocalization between ATG9A and LC3, ATG9A and F-actin, ATG9A and EEA1, ATG9A and RAB11 following different treatments (siRNA or drugs). To have a more precise idea about the colocalization between two markers (the percentage of pixels of one marker that overlaps with another marker), we used the Mander's coefficient.

### Proximity ligation assay

The proximity ligation assay kit was obtained from Sigma and used according to manufacturer's instructions. Briefly, cells were seeded on 13 mm coverslips and allowed to grow in culture for 24 h. After fixation with ice-cold methanol, and blocking with 10% fetal bovine serum in PBS, the cells were incubated with anti-Annexin A2 (Santa Cruz Biotechnology; 3D5) and anti-ATG9A (Abcam; EPR2450(2)). Following incubation with the primary antibodies, the cells were incubated with secondary antibodies conjugated to oligonucleotide primers. The primers were ligated and then rolling circle amplification was used to create a reaction product that is observable by microscopy due to hybridization of fluorescently labelled nucleotides. Successful production of a DNA product requires that the primary antibodies bind their respective antigens and reside within 40 nm of each other. Coverslips were mounted on slides and imaged by confocal microscopy.

### Transferrin recycling assay

Transferrin recycling assay was performed as previously described^58^. Briefly, the cells were incubated for 30 min at 37 °C in the continuous presence of transferrin–Alexa-Fluor-647 (0.05 mg ml^−1^). Cells were then washed and incubated at 37 °C in media supplemented with 0.2 mg ml^−1^ unlabelled transferrin for various times before fixation in 4% paraformaldehyde in PBS. Cell-associated transferrin–Alexa-Fluor-647 was determined by FACS analysis using BD FACS Calibur flow cytometer (BD Biosciences) and FlowJo software (Tree Star Inc.).

### Automatic vesicle tracking

Analysis of ATG9A-GFP vesicle movement was performed using Imaris software and the automatic particle-tracking programme. Imaris provides statistical data that is specific to tracking such as length of tracks and speed. The data originated from Imaris, track length (μm) were plotted on graphs using Excel and analysed statistically using Mann–Whitney test.

### Annexin A2 expression by qPCR

Total RNA was first extracted from cells using TRIzol (Invitrogen). Reverse transcription was performed using SuperScript III First-Strand Synthesis System for PCR with reverse transcription (Invitrogen). Annexin A2 specific primers were purchased from Invitrogen (human specific: forward: GCCATCAAGACCAAAGGTGT, reverse: TCAGTGCTGATGCAAGTTCC; mouse specific: forward: ACGCTGGAGTGAAGAGGAAA, reverse: ACAGGGGCTTGTTCTGAATG). Fold change values were calculated using the ΔΔCt method. An unpaired *t*-test was used to calculate statistical significance.

### Chromatin immunoprecipitation

A total of 10^8^ HeLa cells/condition was crosslinked using 1% formaldehyde in growth medium for 10 min and then cells were treated with 0.215 M glycine for 5 min to stop the crosslinking and washed twice with PBS. Cells were lysed in buffer A (10 mM Tris pH 8.0, 10 mM NaCl and 0.2% NP40) supplemented with 10 mM NaBu and protease/phosphatase inhibitors mix (Roche) for 10 min on ice. The nuclei were recovered and resuspended in buffer B (50 mM Tris pH 8.1, 10 mM EDTA and 1% SDS) supplemented with 10 mM NaBu and protease/phosphatase inhibitors mix (Roche) and incubated for 10 min on ice. Cells were then diluted 2 × in buffer C (20 mM Tris pH 8.1, 2 mM EDTA, 150 mM NaCl, 1% Triton X100 and 0.01% SDS) supplemented with 10 mM NaBu and protease/phosphatase inhibitors mix (Roche) before sonication for 10 min at 4 °C. Chromatin was then cleared and equal amounts were incubated overnight at 4 °C on a rotating wheel with anti-c-Jun antibody—ChIP Grade (Abcam; ab31419), anti-Histone H3 (Abcam; ab8580) and anti-mouse IgG produced in rabbit (Sigma; M7023). Immunocomplexes were isolated using protein A-sepharose (GE Healthcare), washed twice with buffer D (20 mM Tris pH 8.1, 2 mM EDTA, 50 mM NaCl, 1% Triton X100 and 0.1% SDS) and once with buffer E (10 mM Tris pH 8.1, 1 mM EDTA, 0.25 M LiCl, 1% NP40 and 0.1% sodium deoxycholate monohydrate) and finally once with TE buffer. Samples were then eluted using buffer F (100 mM NaHCO_3_ and 1% SDS). The crosslinking was reversed by treating the samples with RNase A and NaCl at a final concentration of 0.3 M overnight at 67 °C and subsequent treatment with proteinase K (Fisher Scientific) for 2 h at 45 °C. Samples were then cleaned using Qiaquick PCR Purification Kit (Qiagen) and subjected to a Real-Time PCR analysis. The primers used for the amplification of c-Jun binding site in Annexin A2 1 promoter are: 5′-CCTGGGTGGGGCTTTTATAC-3′ and 5′-GTGAGTCACCCCTGACTTGG-3′.

### Luciferase reporter assays

HeLa cells were seeded in six multiwells and transfected with 2 μg of the indicated luciferase reporter and cultured in a full medium for 48 h. The luciferase activity was then measured following the manufacturer instructions (Dual luminescence Assay Kit, GeneCopoeia). Point and double mutations within the c-Jun recognition site of the promoter of Annexin A2 were generated with QuikChange Multi Site-Directed Mutagenesis Kit (Agilent Stratagene; 200515).

### Statistical analysis

Significance levels for comparisons between groups were determined with *t*- tests, repeated measure, factorial ANOVA or Mann–Whitney using the STATVIEW software, version 4.53 (Abacus Concepts, Berkeley, CA).

## Additional information

**How to cite this article:** Moreau, K. *et al.* Transcriptional regulation of Annexin A2 promotes starvation-induced autophagy. *Nat. Commun.* 6:8045 doi: 10.1038/ncomms9045 (2015).

## Supplementary Material

Supplementary Figures Supplementary Figures 1-5

Supplementary Movie 1 Live cell imaging of ATG9A-GFP and actin-mRFP. HeLa cells transfected for 20 h with ATG9A-GFP and actin-mRFP were subjected to live cell imaging. Time series: 30 frames/sec for 5 min.

Supplementary Movie 2 Live cell imaging of ATG9A-GFP and mRFP-RAB5. HeLa cells transfected for 20 h with ATG9A-GFP and mRFP-RAB5 were subjected to live cell imaging. Time series: 30 frames/sec for 5 min.

Supplementary Movie 3 Live cell imaging of ATG9A-GFP and mRFP-RAB5 – CK-666 treatment. HeLa cells transfected for 20 h with ATG9A-GFP and mRFP-RAB5 were subjected to live cell imaging after treatment with CK-666 for 2 h. Time series: 30 frames/sec for 5 min.

Supplementary Movie 4 Live cell imaging of ATG9A-GFP and mRFP-RAB5 – Jasplakinolide treatment. HeLa cells transfected for 20 h with ATG9A-GFP and mRFP-RAB5 were subjected to live cell imaging after treatment with Jasplakinolide for 2 h. Time series: 30 frames/sec for 5 min.

Supplementary Movie 5 Live cell imaging of ATG9A-GFP and mRFP-RAB5 – Control. HeLa cells transfected for 20 h with ATG9A-GFP and mRFP-RAB5 and subjected to live cell imaging. Time series: 30 frames/sec for 5 min.

Supplementary Movie 6 Live cell imaging of ATG9A-GFP and mRFP-RAB5 – Annexin A2 knockdown. HeLa cells with Annexin A2 knockdown for 48h using Annexin A2 siRNA were transfected for 20 h with ATG9A-GFP and mRFP-RAB5 and subjected to live cell imaging. Time series: 30 frames/sec for 5 min.

Supplementary Movie 7 Live cell imaging of ATG9A-GFP and mRFP-RAB5 – ARP2 knockdown. HeLa cells with ARP2 knockdown 48h using ARP2 siRNA were transfected for 20 h with ATG9A-GFP and mRFP-RAB5 and subjected to live cell imaging. Time series: 30 frames/sec for 5 min.
