# Supplementary material for: Transcriptional regulation of Annexin A2 promotes starvation-induced autophagy
Source: Nat Commun. 2015 Aug 20;6:8045. doi: 10.1038/ncomms9045 (PMC4560779; doi:10.1038/ncomms9045)
Supplement: Supplementary Figures — 1-5 [file ncomms9045-s1.pdf]

Supplementary Figure 1: Annexin A2 regulates autophagy

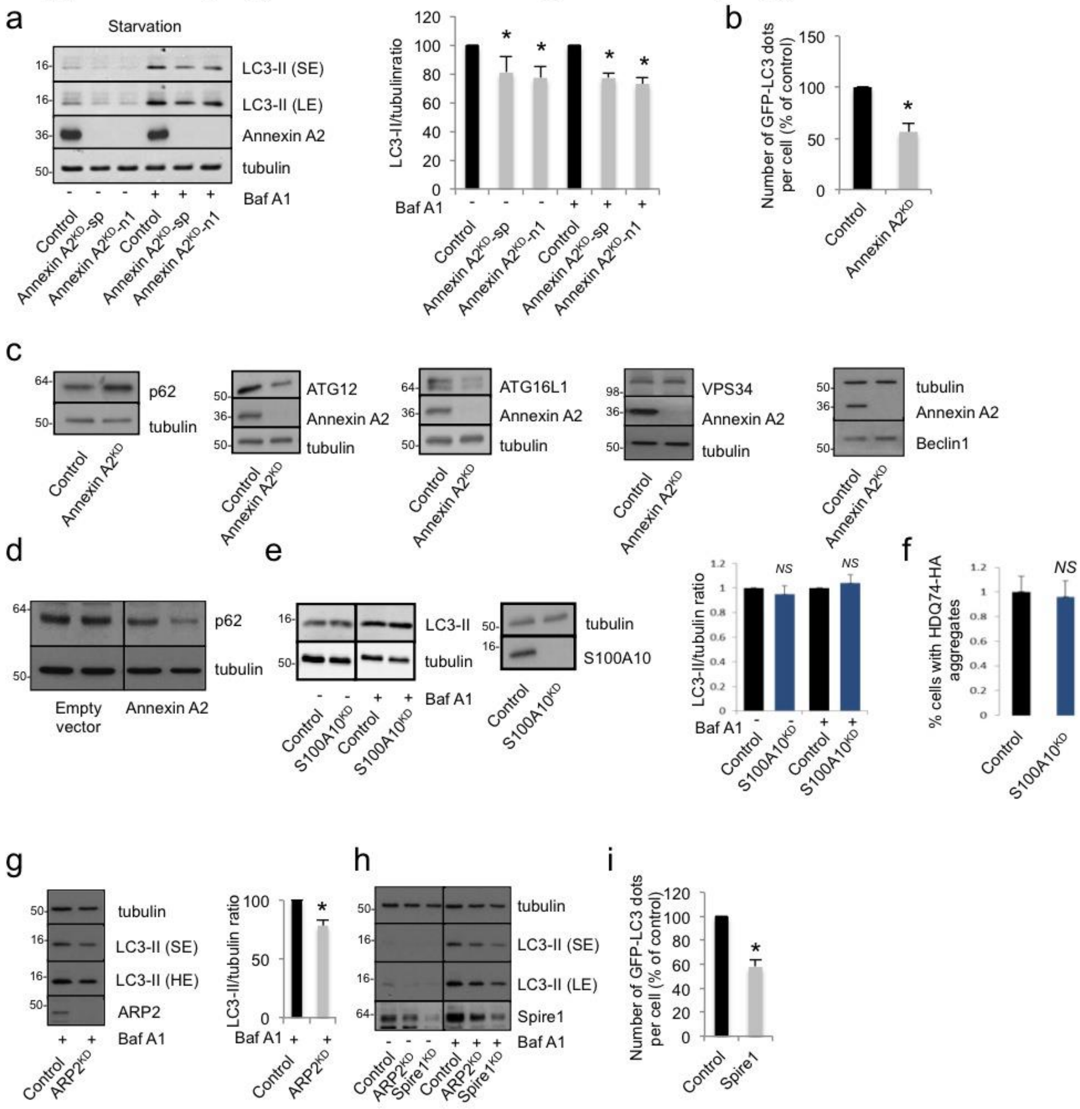

Supplementary Figure 1. Annexin A2 regulates autophagy.

(a). Western blot analysis of tubulin, Annexin A2 and LC3-II in HeLa where Annexin A2 was knocked down using different siRNA (sp: Smartpool; n1: oligo number 1 from the Smartpool). The cells were starved in HBSS and treated with bafilomycin A1 (Baf A1) as indicated. (SE: short exposure; LE: longer exposure). Quantification of LC3-II/tubulin is shown. Data are mean +/- SEM (n = 3 experiments; *: $p<0.05$; two tail *t*-test).

(b). Number of GFP-LC3 dots per cell in Annexin A2 knockdown. HeLa cells where Annexin A2 was knocked down were fixed and subjected to automated microscopy. The data represent the number of LC3 dots per cell shown as mean +/- S.D. (*: $p<0.05$; two-tailed *t*-test; n ≥ 200 cells per condition).

(c). Western blot analysis of tubulin, ATG12, Annexin A2, ATG16L1, VPS34, Beclin1 and p62 in HeLa cells where Annexin A2 was knocked down using siRNA.

(d). Western blot analysis of tubulin and p62 in HeLa cells where Annexin A2 was transiently expressed for 24h.

(e). Western blot analysis of tubulin, S100A10 and LC3-II in HeLa where S100A10 was knocked down using siRNA. The cells were treated with bafilomycin A1 (Baf A1) as indicated. Quantification of LC3-II/tubulin ratio is shown as mean +/- SD (NS: not significant).

(f). Quantification the proportion of mutant huntingtin (Q74)-expressing cells with aggregates (HA-HDQ74) in S100A10 knockdown cells. HeLa cells transiently expressing HA-HDQ74 were fixed and subjected to microscopy after HA-HDQ74 immunostaining using an anti-HA specific antibody. Data are mean +/- SD of the percentage of cells with HA-HDQ74 aggregates (n = 3 experiments; *: $p<0.05$; two tail *t*-test).

(g). Western blot analysis of tubulin, ARP2 and LC3-II in HeLa where ARP2 was knocked down using siRNA. The cells were treated with bafilomycin A1 (Baf A1) as indicated. (SE: short exposure; LE: longer exposure). Quantification of LC3-II/tubulin ratio is shown as mean +/- SD.

(h). Western blot analysis of tubulin, Spire1 and LC3-II in HeLa where Spire1 and ARP2 were knocked down using siRNA. The cells were treated with bafilomycin A1 (Baf A1) as indicated. (SE: short exposure; LE: longer exposure).

(i). Number of GFP-LC3 dots per cell in Spire1 knockdown. HeLa cells where Spire1 was knocked down were fixed and subjected to automated microscopy. The data represent the number of LC3 dots per cell shown as mean +/- S.D. (*: $p<0.05$; two-tailed t-test; n ≥ 200 cells per condition).

Supplementary Figure 2: actin and Annexin A2 localization to ATG9A vesicles

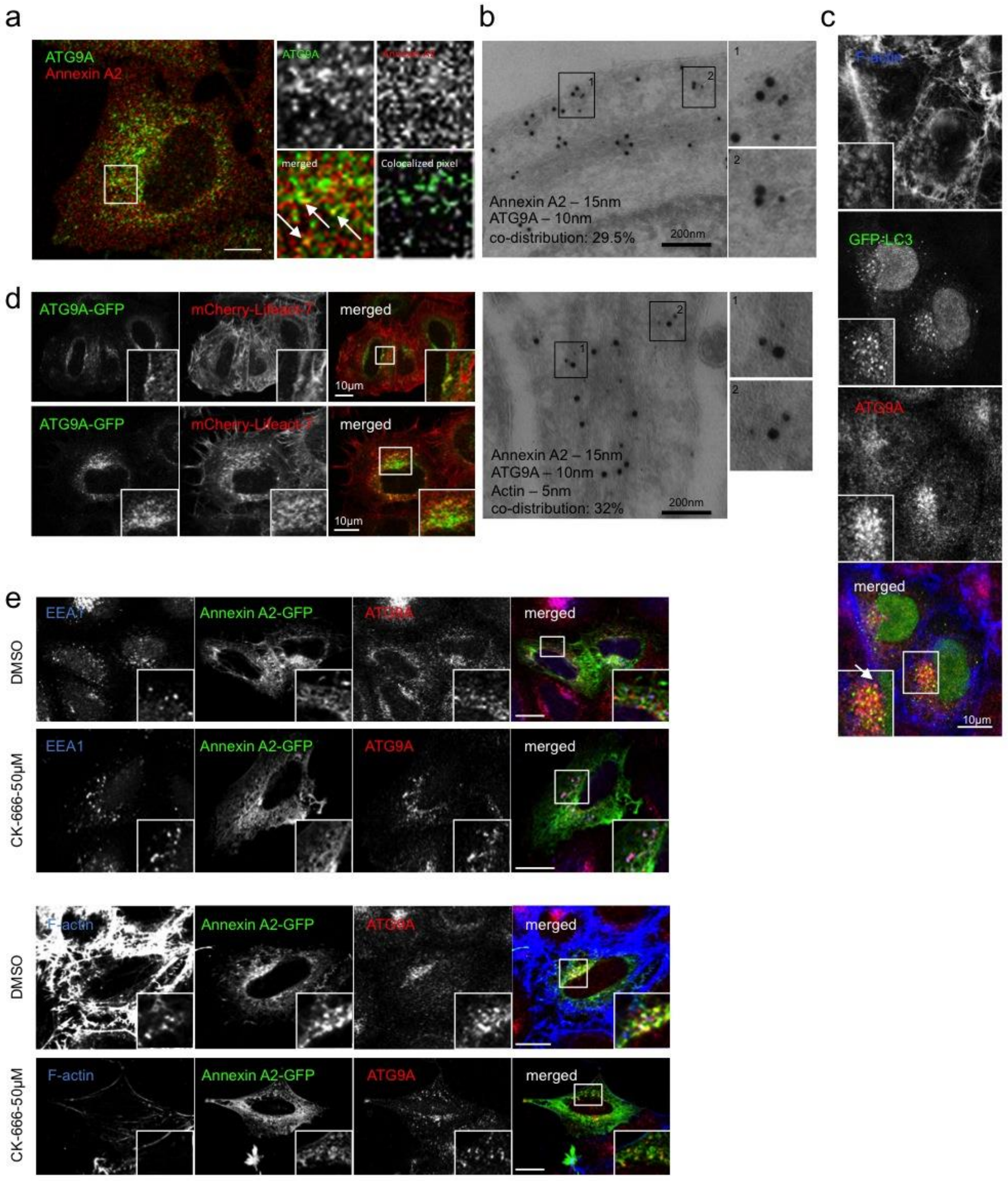

Supplementary Figure 2. Actin and Annexin A2 localization to ATG9A vesicles.

(a). Colocalization between endogenous ATG9A and endogenous Annexin A2 in HeLa cells. Confocal pictures are shown with magnified areas. Arrows indicate ATG9A vesicles positive for Annexin A2. See bottom right magnified panel to visualise colocalised pixels. Scale bars, 5 µm.

(b). Electron microscopy analysis of ATG9A, Annexin A2 and actin distribution. HeLa cells were fixed and treated for immunogold labelling on cryosections. ATG9A was detected with anti-ATG9A antibody (10nm), Annexin A2 was detected with anti-Annexin A2 antibody (15nm) and actin was detected with anti-actin antibody (5nm). 29.5% of Annexin A2 gold particles co-distributed with ATG9A gold particles and 32% of ATG9A gold particles co-distributed with actin gold particles.

(c). Colocalization between ATG9A, GFP-LC3 and F-actin (using phalloidin staining) in HeLa cells. Confocal pictures are shown with magnified areas. Arrow indicates an ATG9A vesicle positive for GFP-LC3 and actin.

(d). Colocalization between ATG9A and actin (using Lifeact-7 expression construct) in HeLa cells. Confocal pictures showing colocalization between ATG9A and actin are presented with magnified areas.

(e). Colocalization between ATG9A, Annexin A2-GFP and EEA1 or F-actin (using Phalloidin staining) in HeLa treated with CK-666 (50µM) drug affecting actin polymerization via ARP2/3 inhibition for 2h. Confocal pictures showing colocalization between ATG9A, Annexin A2-GFP and EEA1 or F-actin are presented with magnified areas.

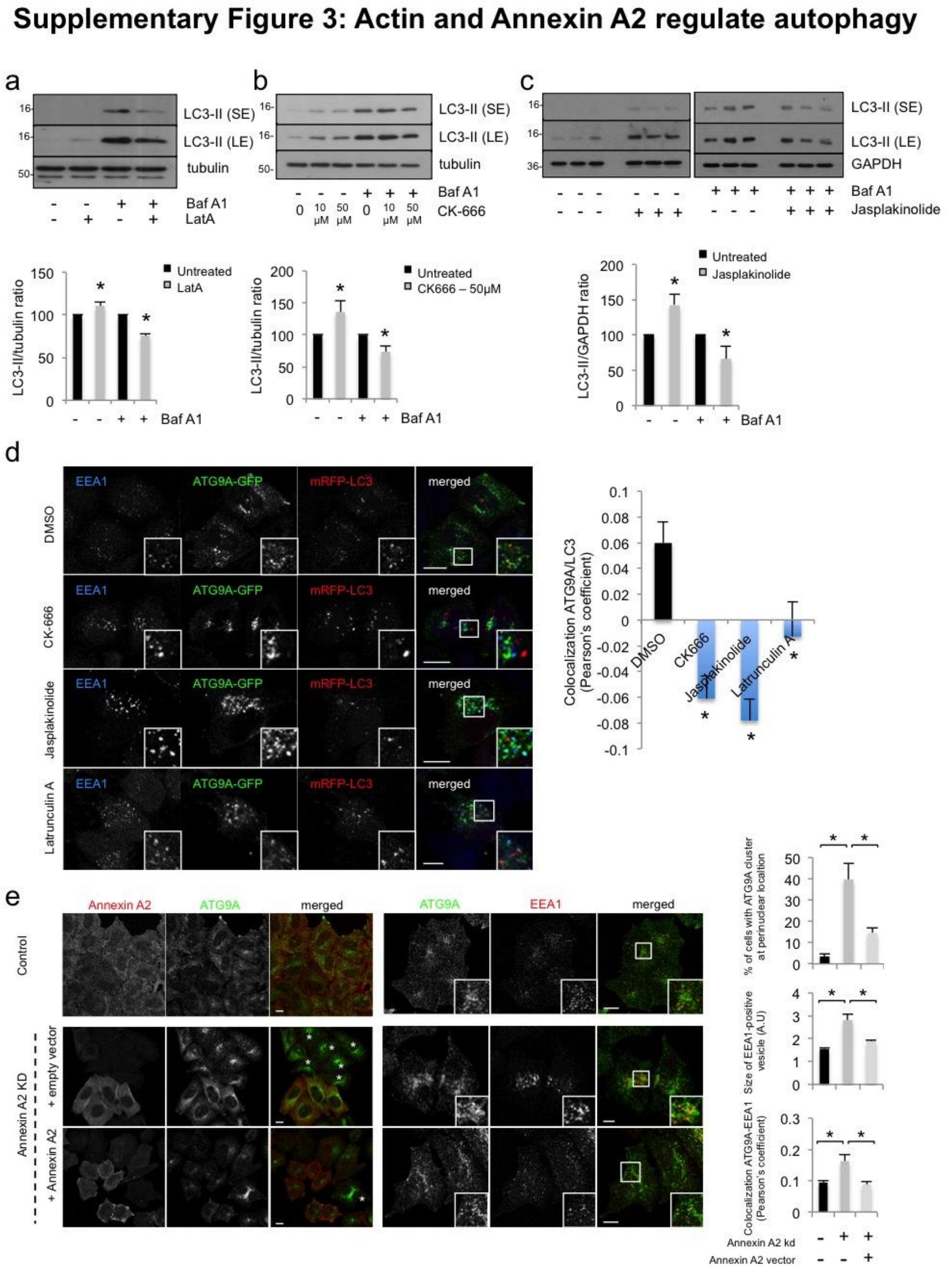

Supplementary Figure 3. Actin and Annexin A2 regulate autophagy.

(a-c). Western blot analysis of LC3-II and tubulin or GAPDH in HeLa cells treated with latrunculin A (LatA, 1µM for 1h), jasplakinolide (200nM for 4h), CK-666 (50µM for 4h) and bafilomycin A1 (Baf A1, 400nM for 4h), as indicated. Data are mean +/- SD (n = 3 experiments; *: $p<0.05$; two tail *t*-test).

(d). Colocalization between ATG9A-GFP, mRFP-LC3 and EEA1 in HeLa treated with drugs affecting actin polymerization for 2h; latrunculin A (0.5µM), jasplakinolide (200nM) and CK-666 (50µM) (top panels). Confocal pictures showing colocalization between ATG9A-GFP, EEA1 and mRFP-LC3 are presented with magnified areas. Quantification of ATG9A and LC3 colocalization is shown on the right as Pearson's coefficient. Data shown as mean +/- SEM ($n \geq 20$ cells; *: $p<0.05$; two tail *t*-test).

(e). Colocalization between ATG9A, EEA1 and Annexin A2 in Annexin A2 knockdown and/or transiently expressing cells. Confocal pictures showing colocalization between ATG9A, EEA1 and Annexin A2 are presented with magnified areas. * indicate Annexin A2 knockdown cells with ATG9A clustering close to perinuclear location. Quantification of the percentage of cells with ATG9A cluster at perinuclear location, size of EEA1-positive vesicle and ATG9A and EEA1 colocalization is shown at the bottom. Data shown as mean +/- SEM ($n \geq 20$ cells; *: $p<0.05$; two tail *t*-test). Scale bars, 5 µm.

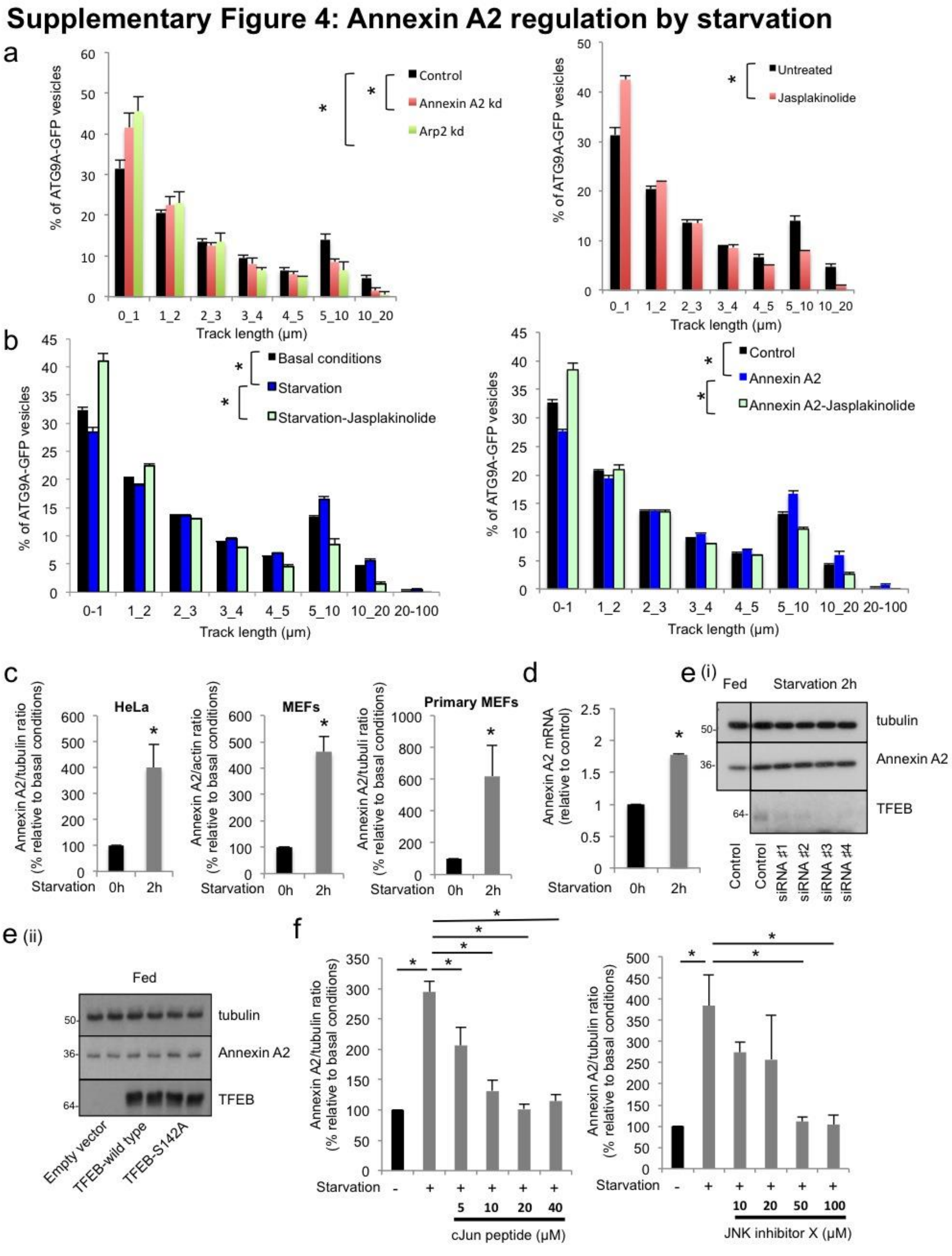

Supplementary Figure 4. Annexin A2 regulation by starvation.

(a). Movement of ATG9A vesicles in Annexin A2 and ARP2 knockdown cells. Cells transiently expressing ATG9A-GFP were treated with Jasplakinolide as indicated, and subjected to live cell imaging. Movement of ATG9A-GFP was quantified using automatic particle tracking. Data are mean +/- SEM of the percentage of ATG9A-

GFP vesicles per track length in µm (n > 200 vesicles; *: $p<0.05$; Mann-Whitney: movies were recorded for 5 min for each condition).

(b). Movement of ATG9A vesicles upon starvation and Annexin A2 overexpression. Cells transiently expressing ATG9A-GFP (left panel) or both ATG9A-GFP and Annexin A2 (right panel) were starved in HBSS for 2h or treated with Jasplakinolide (200nM) as indicated, and subjected to live cell imaging. Movement of ATG9A-GFP was quantified using automatic particle tracking. Data are mean +/- SEM of the percentage of ATG9A-GFP vesicles per track length in µm (n > 200 vesicles; *: $p<0.05$; Mann-Whitney: movies were recorded for 5 min for each condition).

(c). Quantification of the experiments described in Figure 5A. Data are mean +/- SD of Annexin A2/loading control (tubulin, GAPDH or actin) ratio (n = 3 experiments; *: $p<0.05$; two tail one sample *t*-test).

(d). Expression of Annexin A2 quantified by qPCR in HeLa cells. The cells were starved in HBSS as indicated. Data are mean +/- SD of Annexin A2 mRNA (relative to control gene: GAPDH) (n = 3 experiments; *: $p<0.05$; two tail one sample *t*-test).

(e). Western blot analysis of Annexin A2, TFEB (endogenous, TFEB-FLAG wild type and S142A mutant) and tubulin in HeLa cells knockdown for TFEB using differents siRNAs (i) or transiently expressing TFEB (ii). Cells were starved for 2h in HBSS solution.

(f). Quantification of the experiments described in Figure 5C. Data are mean +/- SD of Annexin A2/tubulin ratio (n = 3 experiments; *: $p<0.05$; two tail one sample *t*-test).

Supplementary Figure 5: uncropped scans

From Figure 1A_basal conditions

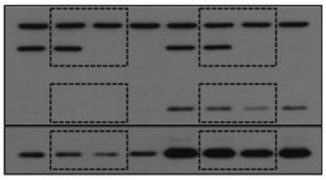

From Figure 1A_starvation

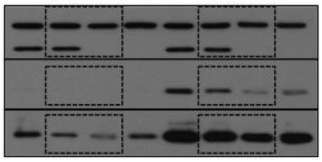

From Figure 5A_MEF ATG16L1 knockout

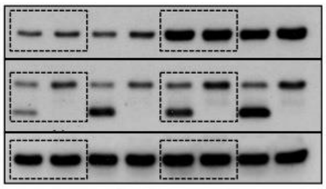

From Figure 5A_primary MEF

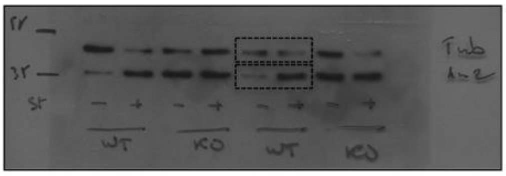

From Figure 5C_cJun peptide

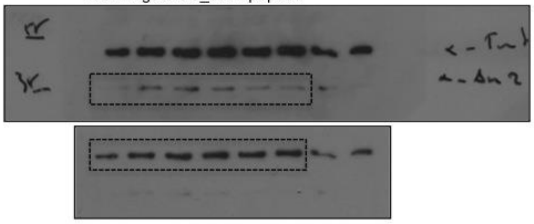

From Figure 5C_JNK inhibitor X

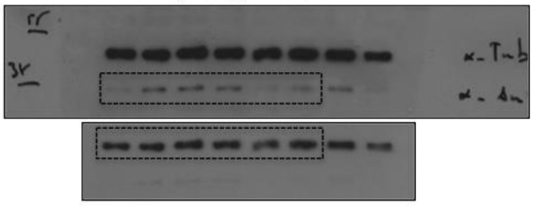

From Figure 5C_cJun

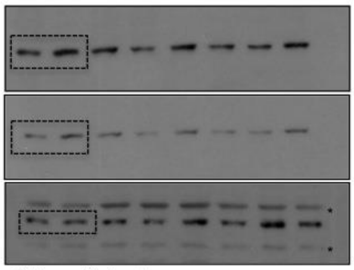

* Unspecific bands

From Figure 5C_JNK

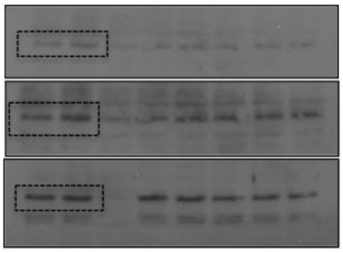

Supplementary Figure 5. Uncropped scans.
